# Elucidating molecular mechanisms of acquired resistance to BRAF inhibitors in melanoma using a microfluidic device and deep sequencing

**DOI:** 10.5808/gi.20074

**Published:** 2021-03-15

**Authors:** Jiyeon Han, Yeonjoo Jung, Yukyung Jun, Sungsu Park, Sanghyuk Lee

**Affiliations:** 1 Department of Bio-information Science, Ewha Womans University, Seoul 03760, Korea; 2 Ewha Research Center for Systems Biology (ERCSB), Ewha Womans University, Seoul 03760, Korea; 3 Center for Supercomputing Application, Division of National Supercomputing, Korea Institute of Science and Technology Information, Daejeon 34141, Korea; 4 School of Mechanical Engineering, Sungkyunkwan University, Suwon 16419, Korea

**Keywords:** cancer drug resistance, melanoma, microfluidic device, RNA sequencing, targeted therapy, whole exome sequencing

## Abstract

BRAF inhibitors (e.g., vemurafenib) are widely used to treat metastatic melanoma with the *BRAF* V600E mutation. The initial response is often dramatic, but treatment resistance leads to disease progression in the majority of cases. Although secondary mutations in the mitogen-activated protein kinase signaling pathway are known to be responsible for this phenomenon, the molecular mechanisms governing acquired resistance are not known in more than half of patients. Here we report a genome- and transcriptome-wide study investigating the molecular mechanisms of acquired resistance to BRAF inhibitors. A microfluidic chip with a concentration gradient of vemurafenib was utilized to rapidly obtain therapy-resistant clones from two melanoma cell lines with the *BRAF* V600E mutation (A375 and SK-MEL-28). Exome and transcriptome data were produced from 13 resistant clones and analyzed to identify secondary mutations and gene expression changes. Various mechanisms, including phenotype switching and metabolic reprogramming, have been determined to contribute to resistance development differently for each clone. The roles of microphthalmia-associated transcription factor, the master transcription factor in melanocyte differentiation/dedifferentiation, were highlighted in terms of phenotype switching. Our study provides an omics-based comprehensive overview of the molecular mechanisms governing acquired resistance to BRAF inhibitor therapy.

## Introduction

Melanoma is a malignant skin cancer that is primarily caused by excessive exposure to UV radiation from sunlight. Although melanoma represents a small proportion of skin cancer, its metastatic form is fatal, with a 5-year survival rate between 5%‒19% [1]. In 2020, more than 100,000 new melanoma patients and 6,850 deaths from melanoma are expected in the United States [2]. Melanoma has been the focus of modern genomic studies and cancer therapeutics since the development of targeted cancer drugs and immunotherapies.

*BRAF* mutation occurs in more than 80% of melanoma patients, with the V600E mutation being the most frequently observed [3]. It is also responsible for ~40% of papillary thyroid carcinoma and small portion of other tumors (e.g., colon, pancreas, brain, lung, etc.), taking ~8% of human tumors in total [4,5]. Vemurafenib, targeting *BRAF* V600 alterations, is among the most well-known cancer drugs with rapid and dramatic early responses, but the tumor eventually relapses in most cases [6]. Thus, overcoming resistance to targeted therapy is of prime importance and would require a detailed understanding of the molecular mechanisms underlying resistance development. Intrinsic tumor heterogeneity and the evolution of cancer cells are the major causes of therapy resistance [7].

Molecular mechanisms of resistance to BRAF inhibitor (BRAFi) have been reported by analyzing the genome and transcriptome data from patient samples [8,9]. Reactivation of the mitogen-activated protein kinase (MAPK) pathway by secondary mutations in the RAS/RAF/MEK/ERK signaling cascade is the most frequently observed mechanism, occurring in up to 80% of BRAFi-resistant tumors [10]. Many additional mechanisms, however, have been found to contribute to the development of therapy resistance, including activation of the phosphoinositide 3-kinase (PI3K)‒AKT‒mammalian target of rapamycin (mTOR) pathway, tumor microenvironment reprogramming by the Hippo signaling pathway, and phenotype switching by master transcription factors (TFs), such as microphthalmia-associated transcription factor (MITF), and receptor tyrosine kinase (RTK), such as AXL [11].

Although patient tumor samples are highly useful for their clinical relevance, it is difficult to study the details of molecular mechanisms because of various issues in sample quality and quantity. Therefore, patient-derived cell lines are important for studying the complex interplay among various mechanisms. In the case of cell line studies for drug resistance, the most difficult and labor-intensive part usually is obtaining resistant cells. Previously, we demonstrated that a microfluidic chip with a concentration gradient of cancer drugs could induce drug resistance rapidly, thereby being designated as the cancer drug resistance accelerator (CDRA) chip, and that multiple molecular mechanisms underlying erlotinib resistance could be elucidated by analyzing exome and transcriptome sequencing data [12]. In this study, we applied the same principle to investigate the mechanisms governing the resistance of melanoma cells to the BRAFi vemurafenib. The aims of our study were not only to identify resistance mechanisms but also to investigate whether different clones or cell lines acquire drug resistance in different ways, i.e., whether the resistance acquisition process is stochastic.

## Methods

### Cell culture and establishment of vemurafenib-resistant cells

Human melanoma cell lines A375 and SK-MEL-28 were purchased from the American Type Culture Collection (Manassas, VA, USA) and were maintained in Dulbecco's modified Eagle's medium (for A375) or minimum essential medium (for SK-MEL-28) supplemented with 10% fetal bovine serum (HyClone, Logan, UT, USA), 100 units/mL penicillin (Invitrogen, Carlsbad, CA, USA) and 100 µg/mL streptomycin (Invitrogen).

Vemurafenib-resistant A375 and SK-MEL-28 cells were established using a microfluidic chip as described previously [12]. Briefly, the interior surface of the chip was sanitized with 70% ethanol and coated with 10 µg/mL fibronectin (Sigma, St. Louis, MO, USA). The cells were seeded carefully onto the surface of a chip and incubated to adhere to the surface. One inlet reservoir was filled with serum-free media containing vemurafenib, and the other inlet reservoir was filled with serum-free media only (i.e., without vemurafenib). Two outlet reservoirs were filled with serum-free media. The reservoirs were replaced every day with freshly prepared serum-free media with or without vemurafenib. After cultivation in the chip, the cells were trypsinized, collected, and transferred to a new culture dish and cultured to obtain enough cells. To examine the effect of exposure to increasing concentrations (0.0001 μM to 10 μM) of vemurafenib (Selleck Chemicals, Houston, TX, USA), cell proliferation was measured at 72 hours using an EZ-Cytox Cell Viability Assay Kit (Daeillab Service, Seoul, Korea). Cells were plated at densities of 4,000 cells or 5,000 cells per well into 96-well plates by hexa-repeat. The results are expressed as a percentage of the cell number in drug-untreated control wells. The IC_50_ values for vemurafenib were calculated by fitting the plot of percentage inhibition versus the log of drug concentration with the nonlinear regression method in GraphPad Prism 6 (GraphPad Inc., La Jolla, CA, USA) software. Error bars represent the standard error of the mean.

### Production and processing of exome and transcriptome sequencing data

Total genomic DNA was extracted from control cells and vemurafenib-resistant cells by the traditional phenol extraction method. One microgram of genomic DNA was used for exome sequencing. Total RNA was extracted from individual conditions using an RNeasy Mini Kit according to the manufacturer’s protocol (Qiagen, Hilden, Germany), and 1 µg of total RNA was used for RNA sequencing. The exome-seq and RNA-seq data acquisition process have been described previously [12].

Sequencing reads were trimmed with Sickle (ver. 1.33) to remove low-quality reads and adaptor sequences [13]. For the RNA sequencing data, trimmed reads were mapped to the reference human genome (UCSC hg19) using STAR (ver. 2.6.1c) [14], and the gene expression values were quantified with RSEM (ver. 1.3.3) [15] with the default options. Exome sequencing data were mapped with the BWA-MEM alignment tool (ver. 0.7.17) [16] and indexed with Samtools (ver. 1.11) [17]. Strelka2 (ver. 2.9.10) [18], as well as Mutect2 [19], in the Genome Analysis Toolkit (GATK ver. 4.1.8.1) were used for calling somatic variants (single nucleotide variations and indels). We also calculated copy number variations (CNVs) from exome sequencing data using EXCAVATOR2 (ver. 1.1.2) [20]. All resulting variants were annotated with ANNOVAR software (ver. 2019Oc24) [21].

### Transcriptome data analysis

Most of our analysis was performed with R (ver. 4.0.0) and several R-based packages. To obtain subgroups from the samples, variable genes were selected within the top 20% in the coefficient of variation (COV20). Hierarchical clustering with these COV20 genes yielded three sample groups, named SK-MEL-28, A375_G1, and A375_G2, according to the cellular origin. Differentially expressed genes were obtained by comparison with the control sample using the edgeR package (ver. 3.30.3) with false discovery rate (FDR) < 0.05, absolute log2FoldChange > 1, and logCPM > 1 [22]. We used the GSVA program (ver. 1.36.3) [23] with the Kyoto Encyclopedia of Genes and Genomes (KEGG) subset of canonical pathways from MSigDB (c2.cp.kegg.v7.2.symbols.gmt) [24] to calculate the gene set activity for each sample. Gene sets with variable activities among three groups were obtained with a t-test supported in R base functions under the threshold of FDR < 0.01.

### Public transcriptome data of melanoma patients

We searched the Gene Expression Omnibus (GEO) for transcriptome data before and after vemurafenib treatment in melanoma patients. We identified such patients from three GEO records with the accession numbers GSE141484 [25], GSE50509 [26], and GSE99898 [27]. Normalized expression data were downloaded and merged with quantile normalization to reduce the batch effect. The fold change in expression was used for heatmap visualization and calculating pathway activities for each sample using the GSVA program. The correlation with the *MITF* expression pattern was calculated with *MITF* and its regulators, RTK genes, and the pathway activities of KEGG pathways. The genes below the correlation coefficient of 0.5 were filtered out. We also performed a principal component analysis using genes highly correlated with *MITF* expression. The R based package factoextra (ver. 1.0.7) was employed to visualize the principal component analysis (PCA) plot of our samples and public patient data.

## Results

### Acquisition of vemurafenib-resistant cells using a microfluidic CDRA chip

**Fig. 1.**
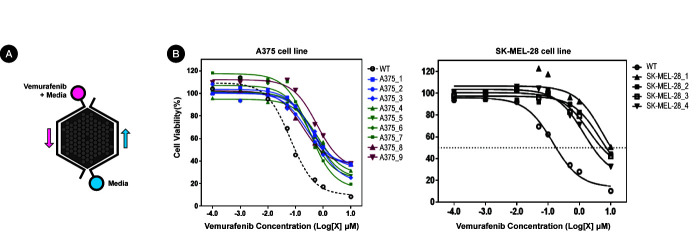
Acquisition of drug-resistant cells using microfluidic chips. (A) Schematic diagram of microfluidic chips and the experimental setup. (B) Cell viability plots to confirm drug resistance (i.e., increased IC_50_ values) of cells obtained from microfluidic chips.

We cultured two melanoma cell lines with the *BRAF* V600E mutation, A375 and SK-MEL-28 specifically, for 8‒47 days on CDRA chips with a concentration gradient of vemurafenib (Fig. 1A). The cultured product was further maintained in dishes with a relatively high dose of vemurafenib to select vemurafenib-resistant cells. We obtained 13 samples, specifically four from SK-MEL-28 and nine from A375 cell lines, and confirmed that those samples were indeed resistant to vemurafenib (Fig. 1B). The extent of resistance, however, was notably different between the two cell lines. The average IC_50_ values of resistant cells from SK-MEL-28 cells increased by 23.7-fold (from 0.143 to 3.3815 μM), whereas those from A375 cells increased by 6.3-fold (from 0.0655 to 0.4133 μM) (Table 1). Thus, the characteristics of drug resistance were highly dependent on the original cellular identity.

### Transcriptome data show different mechanisms of acquired resistance in the two cell lines

In an effort to identify the molecular mechanisms of drug-induced resistance, we performed exome and transcriptome sequencing for two original cell lines and 13 resistant cells obtained from CDRA chips. Exome sequencing data identified only one mutation with known driver potential (HRAS Q61K) in one of the A375-derived resistant cells (Supplementary Fig. 1). Copy number variation profiles were mild in all cases. Thus, we focused on the interpretation of transcriptome data.

**Fig. 2.**
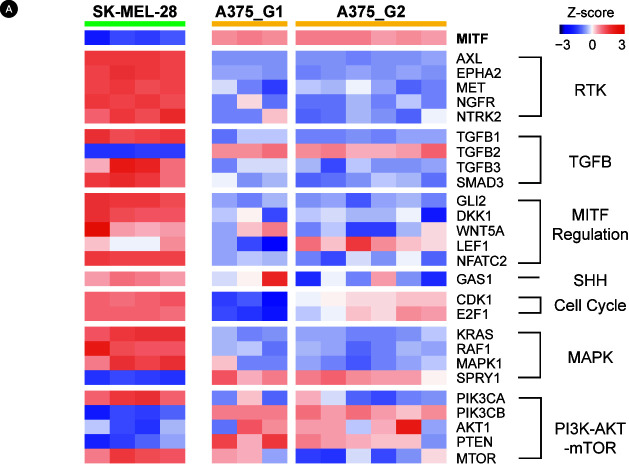
Expression of key genes in drug-resistant A375 and SK-MEL-28 cells. Expression values are the log2FoldChange values converted into the row-wise Z-score. RTK, receptor tyrosine kinase; TGFB, transforming growth factor β; MITF, microphthalmia-associated transcription factor; SHH, smoothened signaling pathway or sonic hedgehog signaling; MAPK, mitogen-activated protein kinase; PI3K, phosphoinositide 3-kinase; mTOR, mammalian target of rapamycin.

Hierarchical clustering of transcriptome data revealed that our resistant cells could be divided into two groups according to the source cell line (Supplementary Fig. 2). Resistant cells from A375 were further divided into two subgroups: A375_G1 and A375_G2. Next, we examined the gene expression of pathway marker genes that are known to be associated with vemurafenib resistance, including the RTK pathway [28], transforming growth factor β (TGF-β) pathway [29], *MITF* regulation [30], Sonic hedgehog pathway [31], MAPK pathway [32-34], and PI3K-AKT-mTOR pathway [11,35] (Fig. 2). The expression levels of most of these known factors were nearly opposite between the SK-MEL-28-derived cells and the A375-derived cells.

Most marker genes showed a dichotomous expression pattern between the SK-MEL-28‒derived cells and A375-derived cells, strongly suggesting that the resistance mechanisms are notably different in the two cell lines. For example, *MITF* and its regulators were downregulated in all SK-MEL-28‒derived cells, while they were upregulated in all A375-derived cells. We also obtained a few markers (e.g., *LEF1*, *CDK1*, *E2F1*, *MAP2K1*, *MAPK3*, *AKT3*, and *MTOR*) showing differences between A375_G1 and A375_G2 cells. Thus, the resistance mechanisms may vary even within the same cell origin, implicating the stochastic nature of the resistance acquisition process.

**Fig. 3.**
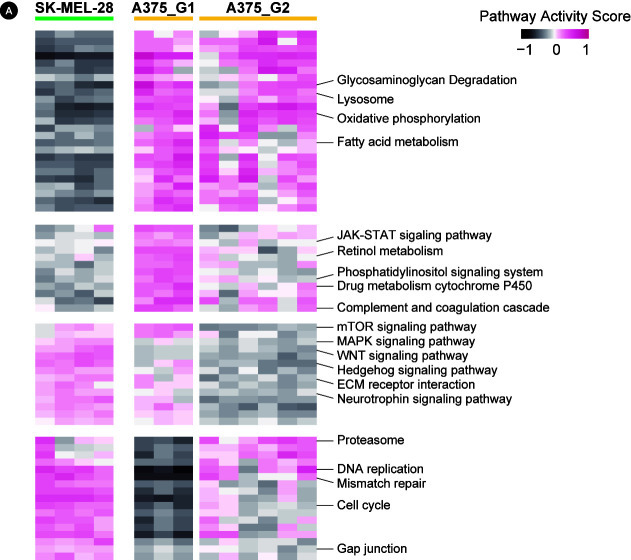
Pathway activities for Kyoto Encyclopedia of Genes and Genomes (KEGG) pathways. The heatmap shows pathway activities for 68 KEGG pathways obtained from the differential test with false discovery rate < 0.05. The full list is shown in Supplementary Table 1 with detailed numbers. mTOR, mammalian target of rapamycin; MAPK, mitogen-activated protein kinase; ECM, extracellular matrix.

To further elucidate resistance mechanisms at the pathway level, we calculated the pathway activities for KEGG curated pathways from MSigDB using the GSVA algorithm (Supplementary Table 1). The pathway activity pattern again showed the same three groups, and differential pathways were obtained by two t-test comparisons of (1) SK-MEL-28‒derived cells and A375-derived cells and (2) A375_G1 cells and A375_G2 cells (Fig. 3).

A number of pathways appeared different between the two cells. SK-MEL-28‒derived cells had downregulated metabolism, upregulated signaling (MAPK, WNT, and Hedgehog pathways), and elevated proliferation (cell cycle, DNA replication, and mismatch repair). A375-derived cells mostly exhibited the opposite expression pattern. The two subgroups of A375 cells had differential activities in mTOR and Hedgehog signaling pathways and RNA homeostasis (basal transcription, spliceosome, and RNA degradation).

### Comparison with clinical samples highlights the roles of MITF and accompanying pathways

Comparison with clinical sample data is critical to *in vitro* experiments, such as the CDRA chip. We collected public expression data of melanoma patients who received vemurafenib treatment because of the V600 mutation and whose transcriptome data were available before and after vemurafenib treatment. We identified 19 such pairs from three studies, enabling multiple pairs to be examined in the case of time series samples [25-27].

**Fig. 4.**
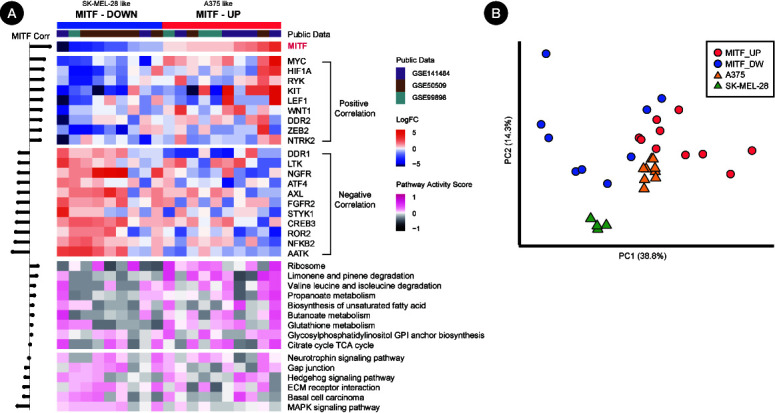
Gene expression and pathway activity from patient samples. (A) Microarray expression data are in log2FoldChange. The expression and pathway activity heatmaps are shown at the top and bottom, respectively, in different colors. Note that the genes and pathways are sorted according to the correlation coefficient with microphthalmia-associated transcription factor (MITF) expression. (B) Principal component analysis plot of both patient samples (circles) and cell line-derived resistant cells (triangles) using 20 correlated genes in A. All expression values are in log2FoldChange.

The regulatory markers in Fig. 2 can be merged into a unifying model where RTK-mediated MAPK and PI3K-AKT pathways affect the expression and nuclear export of MITF [36]. Since MITF is the master regulator of melanocyte proliferation and differentiation as well, we searched for regulatory genes and/or pathways that would play roles within the context of *MITF* regulation. Candidate genes were selected among the RTK genes and known TFs of *MITF* [30,37-39]. Then, correlation coefficients with *MITF* expression were obtained for gene expression or pathway activities using the patient sample pairs (Fig. 4A). Positively correlated genes included *MYC*, *HIF1A*, *RYK*, *KIT*, and *LEF1*, whereas negatively correlated genes were *AATK*, *NFKB2*, *ROR2*, *CREB3*, *STYK1*, *FGFR2*, and *AXL*. We also examined the correlation of pathway activities for KEGG pathways. We used the pathways differentially scored in our data to patient data. As a result, the MAPK pathway and hedgehog pathway showed a high negative correlation tendency, whereas several metabolic pathways had a strong positive correlation. Additionally, cell adhesion-related pathways, such as gap junctions and extracellular matrix (ECM) receptor interactions, showed slight negative correlations.

Since our resistant clones from cell lines and patient samples exhibited dichotomous patterns of *MITF* expression, we performed a PCA of samples using regulator genes. The PCA plot again showed that the two cell lines formed their own clusters, with patient samples scattered more dispersedly but with a specific association with each cell line (Fig. 4B). Thus, our results with cell lines may represent two different classes of patient samples in the molecular mechanism governing the acquisition of resistance to vemurafenib.

## Discussion

Deciphering the molecular mechanisms of resistance is an important step in the development of new treatment methods in targeted cancer therapy. In this study, we adopted a microfluidic chip to rapidly induce drug resistance and applied whole exome and transcriptome sequencing to investigate the molecular mechanisms governing resistance to vemurafenib. Chemo-resistant cell lines are usually acquired by exposing cells to stepwise increasing concentration of chemo-agents, which is labor-intensive and time-consuming. Notably, we obtained resistant cells in 1-7 weeks using the CDRA chips in contrast to several months by conventional methods.

In the case of vemurafenib resistance, genomic variations, such as mutation and CNVs, were relatively mild, probably due to a relatively short time of drug exposure compared to the actual patients. On the other hand, the transcriptome signatures demonstrated that the two cell lines acquired vemurafenib resistance in different ways that are highly contrasted in MITF expression.

MITF is the master TF of melanocytes responsible for regulating the proliferation, differentiation, and metabolic reprogramming of melanocytes. BRAFi treatment causes melanoma cells to change their *MITF* expression to low or high levels, both of which lead to slowly proliferating resistant cells. AXL, an important regulator of apoptosis and epithelial-mesenchymal transition (EMT), is usually inversely correlated with *MITF* expression. Although MITF and AXL have been characterized as two primary regulators of cellular phenotype switching, many other pathways are also relevant to *MITF* regulation, including the MAPK, Hippo, TGF-β, and autophagy signaling pathways. Our pathway activity data showed that many of these pathways were coordinately associated with *MITF* expression in both cell lines and patient tumors, but it was difficult to identify consistent behavior. This finding implies that *MITF* regulation is highly complex and dependent on cellular contexts, such as the tumor microenvironment.

We also observed that A375-derived resistant cells were divided into two subgroups with differential activities in the cell cycle and ECM interactions. These subgroups further highlight the heterogeneity of acquired resistance. Further work, probably based on the systems biology discipline, is necessary to elucidate the roles played by stochastic factors responsible for subgrouping.

Among the regulators and pathways implicated, the MAPK pathway and hedgehog pathways were negatively correlated with *MITF* expression in patients. Hedgehog signaling is known as the master regulator of EMT [40]. AXL and ECM-related pathways further support their role in phenotype transition to induce drug resistance.

Another important factor in phenotype switching is metabolic rewiring. Our data showed that some of the metabolic pathways that were differentially expressed between the A375-derived samples and SK-MEL-28‒derived samples were also positively correlated with *MITF* expression in the patient data. This result is consistent with the findings of a previous report, which asserted that metabolic distractions are the main driver of drug resistance [41,42].

In conclusion, resistance development to vemurafenib treatment is a complex process in which various factors and pathways are involved, including cellular differentiation and dedifferentiation, the tumor microenvironment, and metabolic reprogramming. Further studies to investigate the interplay of these factors and pathways with the master regulator MITF may facilitate the development of new therapies to overcome drug resistance problems in melanoma.

## Figures and Tables

**Table 1.** Experimental conditions

Sample name	Chip-Conc. (μM)	Duration in chip (day)	Dish culture	IC_50_ (μM)	FC to WT
A375 cell line				0.0655	1.0
A375-321s-2	25	13	70 nM (3 wk)	0.3623	5.5
			350 nM (1 wk)		
			3.5 μM (1 wk)		
A375-321s-3	25	13	70 nM (3 wk)	0.2412	3.7
			350 nM (1 wk)		
			3.5μM (1 wk)		
A375-321s-4	25	13	70 nM (3 wk)	0.5420	8.3
			350 nM (1 wk)		
			3.5 μM (1 wk)		
A375-328s-1	10	8	70 nM (3 wk)	0.6387	9.8
			350 nM (1 wk)		
			3.5 μM (1 wk)		
A375-328s-2	10	8	70 nM (3 wk)	0.5076	7.8
			350 nM (1 wk)		
			3.5 μM (1 wk)		
A375-328s-3	10	8	70 nM (3 wk)	0.3329	5.1
			350 nM (1 wk)		
			3.5 μM (1 wk)		
A375-328s-4	10	8	70 nM (3 wk)	0.3190	4.9
			350 nM (1 wk)		
			3.5 μM (1 wk)		
A375-503s-1	10	47	3.5 μM (3 wk)	0.1886	2.9
A375-503s-2	10	47	3.5 μM (4 wk)	0.5874	9.0
SK-MEL-28 cell line				0.143	1.0
SK-MEL-28-1	4.5	12	0.15 μM (6 wk)	5.617	39.3
			1.5 μM (3 wk)		
SK-MEL-28-2	4.5	12	0.15 μM (6 wk)	4.577	32.1
			1.5 μM (3 wk)		
SK-MEL-28-3	7.5	12	0.15 μM (6 wk)	1.966	13.8
			1.5 μM (3 wk)		
SK-MEL-28-4	7.5	12	0.15 μM (6 wk)	1.366	9.6
			1.5 μM (3 wk)		

FC, fold change; WT, wild type.

## Supplementary Materials

Supplementary Table 1. The list of calculated GSVA scores for KEGG curated pathways

Supplementary Fig. 1. Somatic variations from exome sequencing analysis. Mutation plot showing the single nucleotide variants (SNVs), indels, and copy number variations. The genes on the row are from the collection of mitogen-activated protein kinase pathway, receptor tyrosine kinase, and microphthalmia-associated transcription factor (*MITF*) regulations. All SNVs are over the allele frequency of 0.1. The insertion/deletion variations were below the allele frequency of 0.1. Copy number variations (CNVs) were copy number loss events.

Supplementary Fig. 2. Hierarchical clustering of resistant cells. (A) Hierarchical clustering with the top 20% in the coefficient of variation (COV20) genes yielded three sample groups where SK-MEL-28 and A375 cells were divided. A375 cells were further divided into two subgroups. (B) Hierarchical clustering with differentially expressed genes (DEGs) obtained by comparing (1) SK-MEL-28 vs. A375 cells and (2) A375 group 1 vs. A375 group 2 cells. Numbers in parentheses indicate the number of DEGs (upregulated and downregulated in the two comparisons).

## References

[1] Sandru A, Voinea S, Panaitescu E, Blidaru A (2014). Survival rates of patients with metastatic malignant melanoma. J Med Life.

[2] American Cancer Society (2020). Cancer facts and figures.

[3] Davies H, Bignell GR, Cox C, Stephens P, Edkins S, Clegg S (2002). Mutations of the *BRAF* gene in human cancer. Nature.

[4] Alvarez JGB, Otterson GA (2019). Agents to treat *BRAF*-mutant lung cancer. Drugs Context.

[5] Pratilas CA, Xing F, Solit DB (2012). Targeting oncogenic BRAF in human cancer. Curr Top Microbiol Immunol.

[6] Fisher R, Larkin J (2012). Vemurafenib: a new treatment for *BRAF*-V600 mutated advanced melanoma. Cancer Manag Res.

[7] Wagle N, Emery C, Berger MF, Davis MJ, Sawyer A, Pochanard P (2011). Dissecting therapeutic resistance to RAF inhibition in melanoma by tumor genomic profiling. J Clin Oncol.

[8] Romano E, Pradervand S, Paillusson A, Weber J, Harshman K, Muehlethaler K (2013). Identification of multiple mechanisms of resistance to vemurafenib in a patient with *BRAF*V600E-mutated cutaneous melanoma successfully rechallenged after progression. Clin Cancer Res.

[9] Shi H, Hugo W, Kong X, Hong A, Koya RC, Moriceau G (2014). Acquired resistance and clonal evolution in melanoma during BRAF inhibitor therapy. Cancer Discov.

[10] Moriceau G, Hugo W, Hong A, Shi H, Kong X, Yu CC (2015). Tunable-combinatorial mechanisms of acquired resistance limit the efficacy of BRAF/MEK cotargeting but result in melanoma drug addiction. Cancer Cell.

[11] Kozar I, Margue C, Rothengatter S, Haan C, Kreis S (2019). Many ways to resistance: how melanoma cells evade targeted therapies. Biochim Biophys Acta Rev Cancer.

[12] Han J, Jun Y, Kim SH, Hoang HH, Jung Y, Kim S (2016). Rapid emergence and mechanisms of resistance by U87 glioblastoma cells to doxorubicin in an *in vitro* tumor microfluidic ecology. Proc Natl Acad Sci U S A.

[13] Joshi NA, Fass JN (2011). Sickle: a sliding-window, adaptive, quality-based trimming tool for FastQ files (version 1.33).

[14] Dobin A, Davis CA, Schlesinger F, Drenkow J, Zaleski C, Jha S (2013). STAR: ultrafast universal RNA-seq aligner. Bioinformatics.

[15] Li B, Dewey CN (2011). RSEM: accurate transcript quantification from RNA-Seq data with or without a reference genome. BMC bioinformatics.

[16] Li H, Durbin R (2009). Fast and accurate short read alignment with Burrows-Wheeler transform. Bioinformatics.

[17] Li H, Handsaker B, Wysoker A, Fennell T, Ruan J, Homer N (2009). The Sequence Alignment/Map format and SAMtools. Bioinformatics.

[18] Kim S, Scheffler K, Halpern AL, Bekritsky MA, Noh E, Kallberg M (2018). Strelka2: fast and accurate calling of germline and somatic variants. Nat Methods.

[19] Cibulskis K, Lawrence MS, Carter SL, Sivachenko A, Jaffe D, Sougnez C (2013). Sensitive detection of somatic point mutations in impure and heterogeneous cancer samples. Nat Biotechnol.

[20] Magi A, Tattini L, Cifola I, D'Aurizio R, Benelli M, Mangano E (2013). EXCAVATOR: detecting copy number variants from whole-exome sequencing data. Genome Biol.

[21] Wang K, Li M, Hakonarson H (2010). ANNOVAR: functional annotation of genetic variants from high-throughput sequencing data. Nucleic Acids Res.

[22] Robinson MD, McCarthy DJ, Smyth GK (2010). edgeR: a Bioconductor package for differential expression analysis of digital gene expression data. Bioinformatics.

[23] Hänzelmann S, Castelo R, Guinney J (2013). GSVA: gene set variation analysis for microarray and RNA-seq data. BMC Bioinformatics.

[24] Subramanian A, Tamayo P, Mootha VK, Mukherjee S, Ebert BL, Gillette MA (2005). Gene set enrichment analysis: a knowledge-based approach for interpreting genome-wide expression profiles. Proc Natl Acad Sci U S A.

[25] Vergani E, Dugo M, Cossa M, Frigerio S, Di Guardo L, Gallino G (2020). miR-146a-5p impairs melanoma resistance to kinase inhibitors by targeting COX2 and regulating NFκB-mediated inflammatory mediators. Cell Commun Signal.

[26] Rizos H, Menzies AM, Pupo GM, Carlino MS, Fung C, Hyman J (2014). BRAF inhibitor resistance mechanisms in metastatic melanoma: spectrum and clinical impact. Clin Cancer Res.

[27] Kakavand H, Rawson RV, Pupo GM, Yang JY, Menzies AM, Carlino MS (2017). PD-L1 expression and immune escape in melanoma resistance to MAPK inhibitors. Clin Cancer Res.

[28] Easty DJ, Gray SG, O’Byrne KJ, O’Donnell D, Bennett DC (2011). Receptor tyrosine kinases and their activation in melanoma. Pigment Cell Melanoma Res.

[29] Sun C, Wang L, Huang S, Heynen GJ, Prahallad A, Robert C (2014). Reversible and adaptive resistance to *BRAF*(V600E) inhibition in melanoma. Nature.

[30] Goding CR, Arnheiter H (2019). MITF: the first 25 years. Genes Dev.

[31] Li C, Chi S, Xie J (2011). Hedgehog signaling in skin cancers. Cell Signal.

[32] Dietrich P, Kuphal S, Spruss T, Hellerbrand C, Bosserhoff A (2018). Wild-type *KRAS* is a novel therapeutic target for melanoma contributing to primary and acquired resistance to BRAF inhibition. Oncogene.

[33] Sun X, Li J, Sun Y, Zhang Y, Dong L, Shen C (2016). miR-7 reverses the resistance to BRAFi in melanoma by targeting EGFR/IGF-1R/CRAF and inhibiting the MAPK and PI3K/AKT signaling pathways. Oncotarget.

[34] Lito P, Pratilas CA, Joseph EW, Tadi M, Halilovic E, Zubrowski M (2012). Relief of profound feedback inhibition of mitogenic signaling by RAF inhibitors attenuates their activity in BRAFV600E melanomas. Cancer Cell.

[35] Rossi A, Roberto M, Panebianco M, Botticelli A, Mazzuca F, Marchetti P (2019). Drug resistance of *BRAF*-mutant melanoma: review of up-to-date mechanisms of action and promising targeted agents. Eur J Pharmacol.

[36] Ngeow KC, Friedrichsen HJ, Li L, Zeng Z, Andrews S, Volpon L (2018). BRAF/MAPK and GSK3 signaling converges to control MITF nuclear export. Proc Natl Acad Sci U S A.

[37] Hartman ML, Czyz M (2015). MITF in melanoma: mechanisms behind its expression and activity. Cell Mol Life Sci.

[38] Braschi B, Denny P, Gray K, Jones T, Seal R, Tweedie S (2019). Genenames.org: the HGNC and VGNC resources in 2019. Nucleic Acids Res.

[39] Kanehisa M, Goto S (2000). KEGG: kyoto encyclopedia of genes and genomes. Nucleic Acids Res.

[40] Faiao-Flores F, Alves-Fernandes DK, Pennacchi PC, Sandri S, Vicente AL, Scapulatempo-Neto C (2017). Targeting the hedgehog transcription factors GLI1 and GLI2 restores sensitivity to vemurafenib-resistant human melanoma cells. Oncogene.

[41] Ratnikov BI, Scott DA, Osterman AL, Smith JW, Ronai ZA (2017). Metabolic rewiring in melanoma. Oncogene.

[42] Vivas-García Y, Falletta P, Liebing J, Louphrasitthiphol P, Feng Y, Chauhan J (2020). Lineage-restricted regulation of SCD and fatty acid saturation by MITF controls melanoma phenotypic plasticity. Molecular Cell.

