# Supplementary material for: Elucidating molecular mechanisms of acquired resistance to BRAF inhibitors in melanoma using a microfluidic device and deep sequencing
Source: Genomics Inform. 2021 Mar 15;19(1):e2. doi: 10.5808/gi.20074 (PMC8042304; doi:10.5808/gi.20074)
Supplement: Supplementary Table 1. — The list of calculated GSVA scores for KEGG curated pathways [file gi-20074-suppl1.pdf]

Supplementary Table 1. The list of calculated GSVA scores for KEGG curated pathways

Term	Average GSVA score (–1 to 1)				Case 1		Case 2	
	SK-MEL-28	A375	A375_G1	A375_G2	p-value	FDR	p-value	FDR
KEGG_GLYCOSPHINGOLIPID_BIOSYNTHESIS_LACTO_AND_NEOLACTO_SERIES	0.18	0.18	0.12	0.20	6.44E-05	5.99E-04	-	-
KEGG_RENIN_ANGIOTENSIN_SYSTEM	0.27	0.27	0.21	0.30	8.93E-07	5.04E-05	-	-
KEGG_SPHINGOLIPID_METABOLISM	0.24	0.24	0.19	0.27	1.18E-04	9.17E-04	-	-
KEGG_OTHER_GLYCAN_DEGRADATION	0.43	0.43	0.60	0.34	1.08E-07	2.02E-05	-	-
KEGG_GLYCOSPHINGOLIPID_BIOSYNTHESIS_GLOBO_SERIES	0.25	0.25	0.25	0.25	3.28E-05	4.01E-04	-	-
KEGG_GLYCOSYLPHOSPHATIDYLINOSITOL_GPI_ANCHOR_BIOSYNTHESIS	0.22	0.22	0.25	0.21	2.66E-04	1.76E-03	-	-
KEGG_GLUTATHIONE_METABOLISM	0.11	0.11	0.12	0.10	6.39E-04	3.60E-03	-	-
KEGG_GLYCOSAMINOGLYCAN_DEGRADATION	0.27	0.27	0.50	0.15	3.42E-05	4.01E-04	-	-
KEGG_LYSOSOME	0.21	0.21	0.34	0.14	2.93E-06	6.31E-05	-	-

KEGG_ALZHEIMERS_DISEASE	0.19	0.19	0.32	0.12	4.62E-05	4.77E-04	-	-
KEGG_RIBOSOME	0.32	0.32	0.39	0.28	3.05E-05	4.01E-04	-	-
KEGG_OXIDATIVE_PHOSPHORYLATION	0.30	0.30	0.37	0.27	3.06E-06	6.31E-05	-	-
KEGG_PARKINSONS_DISEASE	0.25	0.25	0.30	0.22	5.91E-04	3.55E-03	-	-
KEGG_BIOSYNTHESIS_OF_UNSATURATED_FATTY_ACIDS	0.15	0.15	0.04	0.21	1.57E-03	7.13E-03	-	-
KEGG_ALPHA_LINOLENIC_ACID_METABOLISM	0.19	0.19	0.26	0.16	1.55E-03	7.13E-03	-	-
KEGG_FATTY_ACID_METABOLISM	0.16	0.16	0.36	0.06	7.43E-05	6.58E-04	-	-
KEGG_ARACHIDONIC_ACID_METABOLISM	0.12	0.12	0.24	0.06	6.79E-04	3.72E-03	-	-
KEGG_VALINE_LEUCINE_AND_ISOLEUCINE_DEGRADATION	0.27	0.27	0.40	0.21	5.05E-06	8.54E-05	-	-
KEGG_PROPANOATE_METABOLISM	0.25	0.25	0.38	0.19	2.07E-06	6.31E-05	-	-
KEGG_BUTANOATE_METABOLISM	0.24	0.24	0.26	0.23	1.84E-06	6.31E-05	-	-
KEGG_LIMONENE_AND_PINENE_DEGRADATION	0.26	0.26	0.23	0.27	4.39E-06	8.17E-05	-	-
KEGG_GALACTOSE_METABOLISM	0.20	0.20	0.30	0.15	1.72E-03	7.62E-03	-	-
KEGG_TRYPTOPHAN_METABOLISM	0.18	0.18	0.23	0.16	2.39E-04	1.65E-03	-	-
KEGG_CITRATE_CYCLE_TCA_CYCL	0.21	0.21	0.34	0.14	2.76E-06	6.31E-05	-	-

E								
KEGG_CARDIAC_MUSCLE_CONTRACTION	0.22	0.22	0.33	0.16	1.08E-06	5.04E-05	-	-
KEGG_SNARE_INTERACTIONS_IN_VESICULAR_TRANSPORT	0.03	0.03	0.31	–0.11	8.66E-05	1.34E-03	-	-
KEGG_PROXIMAL_TUBULE_BICARBONATE_RECLAMATION	0.07	0.07	0.19	0.01	9.91E-04	8.02E-03	-	-
KEGG_JAK_STAT_SIGNALING_PATHWAY	0.03	0.03	0.17	–0.04	1.02E-04	1.45E-03	-	-
KEGG_LINOLEIC_ACID_METABOLISM	0.06	0.06	0.32	–0.06	1.87E-05	4.97E-04	-	-
KEGG_RETINOL_METABOLISM	0.07	0.07	0.31	–0.05	3.25E-04	3.56E-03	-	-
KEGG_ABC_TRANSPORTERS	0.09	0.09	0.29	–0.01	4.31E-05	8.49E-04	-	-
KEGG_INOSITOL_PHOSPHATE_METABOLISM	0.05	0.05	0.34	–0.10	1.08E-06	6.70E-05	-	-
KEGG_PHOSPHATIDYLINOSITOL_SIGNALING_SYSTEM	0.08	0.08	0.29	–0.02	8.90E-04	7.52E-03	-	-
KEGG_DRUG_METABOLISM_CYTOCHROME_P450	0.12	0.12	0.35	0.01	1.17E-03	8.73E-03	2.47E-03	9.97E-03
KEGG_PEROXISOME	0.10	0.10	0.29	0.01	4.58E-04	4.48E-03	-	-
KEGG_PORPHYRIN_AND_CHLOROPHYLL_METABOLISM	0.22	0.22	0.37	0.15	1.99E-03	8.61E-03	-	-

KEGG_COMPLEMENT_AND_COAGULATION_CASCADES	0.14	0.14	0.32	0.04	3.10E-04	3.56E-03	-	-
KEGG_MTOR_SIGNALING_PATHWAY	–0.07	–0.07	0.25	–0.23	6.68E-05	6.03E-03	4.97E-05	8.49E-04
KEGG_ENDOMETRIAL_CANCER	–0.02	–0.02	0.19	–0.12	7.32E-04	6.49E-03	-	-
KEGG_MAPK_SIGNALING_PATHWAY	–0.10	–0.10	–0.04	–0.14	6.12E-04	3.56E-03	-	-
KEGG_WNT_SIGNALING_PATHWAY	–0.15	–0.15	–0.12	–0.17	1.02E-03	4.99E-03	-	-
KEGG_BASAL_CELL_CARCINOMA	–0.19	–0.19	–0.11	–0.22	4.44E-07	4.13E-05	-	-
KEGG_HEDGEHOG_SIGNALING_PATHWAY	–0.16	–0.16	0.02	–0.26	5.45E-05	5.33E-04	1.43E-04	6.03E-03
KEGG_TYPE_II_DIABETES_MELLITUS	–0.12	–0.12	0.00	–0.17	4.99E-04	3.09E-03	-	-
KEGG_ECM_RECEPTOR_INTERACTION	–0.11	–0.11	0.04	–0.18	2.37E-03	9.80E-03	-	-
KEGG_ADIPOCYTOKINE_SIGNALING_PATHWAY	–0.08	–0.08	0.08	–0.16	1.55E-04	6.03E-03	-	-
KEGG_NEUROTROPHIN_SIGNALING_PATHWAY	–0.10	–0.10	–0.03	–0.14	2.10E-05	3.25E-04	-	-
KEGG_RENAL_CELL_CARCINOMA	–0.15	–0.15	0.02	–0.24	2.48E-04	6.60E-03	4.59E-04	2.95E-03
KEGG_VASOPRESSIN_REGULATED_WATER_REABSORPTION	–0.15	–0.15	0.12	–0.29	1.64E-04	6.03E-03	-	-
KEGG_CHRONIC_MYELOID_LEUKEMIA	–0.12	–0.12	0.07	–0.21	7.48E-04	3.98E-03	1.32E-06	2.45E-04

KEGG_AXON_GUIDANCE	–0.06	–0.06	0.04	–0.11	1.94E-04	6.03E-03	-	-
KEGG_PROTEASOME	–0.01	–0.01	–0.49	0.24	5.00E-06	1.86E-04	-	-
KEGG_PYRIMIDINE_METABOLISM	–0.02	–0.02	–0.39	0.16	5.02E-05	8.49E-04	-	-
KEGG_RNA_POLYMERASE	–0.01	–0.01	–0.45	0.21	1.14E-03	8.73E-03	-	-
KEGG_BASE_EXCISION_REPAIR	–0.06	–0.06	–0.36	0.09	3.41E-06	1.59E-04	-	-
KEGG_DNA_REPLICATION	–0.13	–0.13	–0.73	0.17	5.45E-07	5.07E-05	-	-
KEGG_MISMATCH_REPAIR	–0.16	–0.16	–0.69	0.11	2.19E-07	4.07E-05	-	-
KEGG_BASAL_TRANSCRIPTION_FACTORS	–0.20	–0.20	–0.38	–0.10	1.66E-04	2.21E-03	1.03E-04	8.68E-04
KEGG_RNA_DEGRADATION	–0.19	–0.19	–0.48	–0.05	2.13E-04	2.64E-03	1.45E-04	1.08E-03
KEGG_SPLICEOSOME	–0.25	–0.25	–0.62	–0.07	9.51E-06	2.95E-04	1.08E-04	8.73E-04
KEGG_CELL_CYCLE	–0.19	–0.19	–0.48	–0.05	2.26E-04	1.61E-03	-	-
KEGG_OOCYTE_MEIOSIS	–0.16	–0.16	–0.35	–0.06	3.67E-05	4.01E-04	4.22E-04	4.36E-03
KEGG_NUCLEOTIDE_EXCISION_REPAIR	–0.16	–0.16	–0.44	–0.02	1.24E-03	5.93E-03	-	-
KEGG_ONE_CARBON_POOL_BY_FOLATE	–0.18	–0.18	–0.42	–0.06	6.23E-04	5.79E-03	9.95E-04	4.99E-03
KEGG_HOMOLOGOUS_RECOMBINATION	–0.15	–0.15	–0.59	0.08	2.37E-05	5.51E-04	-	-
KEGG_DORSO_VENTRAL_AXIS_FORMATION	–0.15	–0.15	–0.27	–0.10	2.20E-03	9.31E-03	-	-
KEGG_GAP_JUNCT	–	–	–	–	3.57E-	4.01E-	-	-
