# Supplementary material for: Elucidating molecular mechanisms of acquired resistance to BRAF inhibitors in melanoma using a microfluidic device and deep sequencing
Source: Genomics Inform. 2021 Mar 15;19(1):e2. doi: 10.5808/gi.20074 (PMC8042304; doi:10.5808/gi.20074)
Supplement: Supplementary Fig. 2. — Hierarchical clustering of resistant cells. (A) Hierarchical clustering with the top 20% in the coefficient of variation (COV20) genes yielded three sample groups where SK-MEL-28 and A375 cells were divided. A375 cells were further divided into two subgroups. (B) Hierarchical clustering with differentially expressed genes (DEGs) obtained by comparing (1) SK-MEL-28 vs. A375 cells and (2) A375 group 1 vs. A375 group 2 cells. Numbers in parentheses indicate the number of DEGs (upregulated and downregulated in the two comparisons). [file gi-20074-suppl3.pdf]

Supplementary Fig. 1. Somatic variations from exome sequencing analysis. Mutation plot showing the single nucleotide variants (SNVs), indels, and copy number variations. The genes on the row are from the collection of mitogen-activated protein kinase pathway, receptor tyrosine kinase, and microphthalmia-associated transcription factor (*MITF*) regulations. All SNVs are over the allele frequency of 0.1. The insertion/deletion variations were below the allele frequency of 0.1. Copy number variations (CNVs) were copy number loss events.

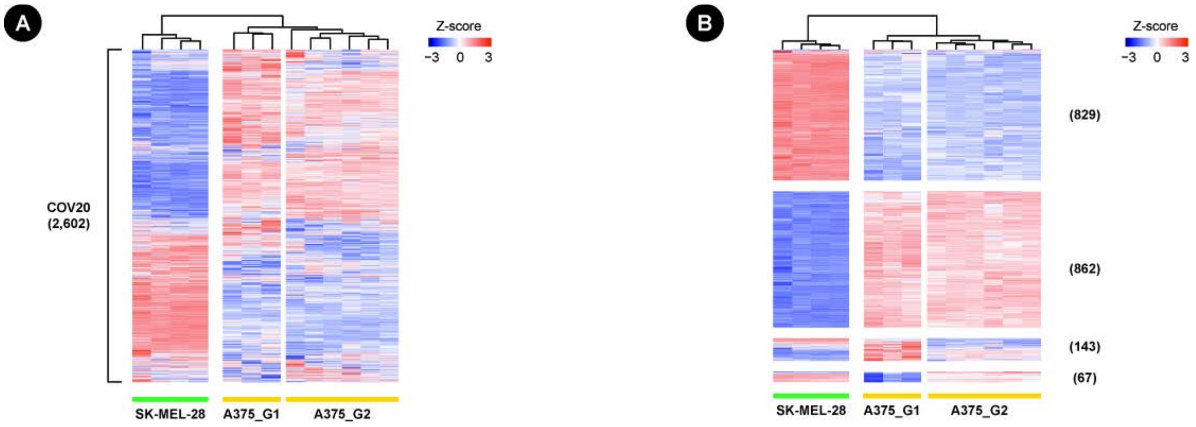

Supplementary Fig. 2. Hierarchical clustering of resistant cells. (A) Hierarchical clustering with the top 20% in the coefficient of variation (COV20) genes yielded three sample groups where SK-MEL-28 and A375 cells were divided. A375 cells were further divided into two subgroups. (B) Hierarchical clustering with differentially expressed genes (DEGs) obtained by comparing (1) SK-MEL-28 vs. A375 cells and (2) A375 group 1 vs. A375 group 2 cells. Numbers in parentheses indicate the number of DEGs (upregulated and downregulated in the two comparisons).
